# Extensive Acclimation in Ectotherms Conceals Interspecific Variation in Thermal Tolerance Limits

**DOI:** 10.1371/journal.pone.0150408

**Published:** 2016-03-18

**Authors:** Anna F. V. Pintor, Lin Schwarzkopf, Andrew K. Krockenberger

**Affiliations:** 1 Centre for Tropical Biodiversity and Climate Change, College of Marine and Environmental Sciences, James Cook University, Cairns, Qld, 4878, Australia; 2 Centre for Tropical Biodiversity and Climate Change, College of Marine and Environmental Sciences, James Cook University, Townsville, Qld, 4811, Australia; University of Sao Paulo, BRAZIL

## Abstract

Species’ tolerance limits determine their capacity to tolerate climatic extremes and limit their potential distributions. Interspecific variation in thermal tolerances is often proposed to indicate climatic vulnerability and is, therefore, the subject of many recent meta-studies on differential capacities of species from climatically different habitats to deal with climate change. Most studies on thermal tolerances do not acclimate animals or use inconsistent, and insufficient, acclimation times, limiting our knowledge of the shape, duration and extent of acclimation responses. Consequently patterns in thermal tolerances observed in meta-analyses, based on data from the literature are based on inconsistent, partial acclimation and true trends may be obscured. In this study we describe time-course of complete acclimation of critical thermal minima in the tropical ectotherm *Carlia longipes* and compare it to the average acclimation response of other reptiles, estimated from published data, to assess how much acclimation time may contribute to observed differences in thermal limits. *Carlia longipes* decreased their lower critical thermal limits by 2.4°C and completed 95% of acclimation in 17 weeks. Wild populations did not mirror this acclimation process over the winter. Other reptiles appear to decrease cold tolerance more quickly (95% in 7 weeks) and to a greater extent, with an estimated average acclimation response of 6.1°C. However, without data on tolerances after longer acclimation times available, our capacity to estimate final acclimation state is very limited. Based on the subset of data available for meta-analysis, much of the variation in cold tolerance observed in the literature can be attributed to acclimation time. Our results indicate that (i) acclimation responses can be slow and substantial, even in tropical species, and (ii) interspecific differences in acclimation speed and extent may obscure trends assessed in some meta-studies. Cold tolerances of wild animals are representative of cumulative responses to recent environments, while lengthy acclimation is necessary for controlled comparisons of physiological tolerances. Measures of inconsistent, intermediate acclimation states, as reported by many studies, represent neither the realised nor the potential tolerance in that population, are very likely underestimates of species’ physiological capacities and may consequently be of limited value.

## Introduction

Climate patterns within species’ geographic distributions shape their physiological traits and tolerances [1–3]. Most notably, expectations for physiological tolerances are expressed in the climatic variability hypothesis, which proposes that species originating from more environmentally variable habitats along a climatic gradient (such as latitude or elevation) evolve broader environmental tolerances and consequently spread to occupy a larger range size along such gradients [1, 4, 5]. Tropical organisms, on the contrary, are expected to have narrower tolerances and limited acclimatisation potential [1, 6].

With respect to latitudinal gradients in climate patterns, the upper limits of temperature variability (i.e. maximum temperatures, and species’ tolerances thereof), have recently received particular attention because of the impending threat of anthropogenic climate change and the prediction of limited capacity of species to deal with rising temperatures [7–9]. However, latitudinal changes in temperature variability arise mainly from geographic variation in minimum temperatures, while maximum temperatures vary comparatively little with latitude [1, 10]. Consequently, differences in tolerance breadth, often assumed to be indicative of species’ overall sensitivity to thermal variability [1], are largely driven by differences in cold tolerances [11, 12]. Adaptations related to tolerance of low temperatures are therefore suggested by some authors to limit current distributions more than heat tolerance and published data is consistent with this [13], especially in ectotherms, because of their limited capacity for active metabolic heat production. Consequently, ectotherms are limited by colder temperatures at high latitudes [14–16] and many species that extend into temperate regions have only achieved these range expansions through mechanisms such as viviparity [14, 17], brumation periods over winter months [18], metabolic compensation or inverse acclimation of metabolic rates at low temperatures [19], lowered critical minimum temperatures or other specialised cold adaptations [20, 21]. Out of these adaptations, tolerances of low temperatures, particularly, appear to show a clear trend across latitude [9, 13, 22, 23], reflecting geographic trends in minimum temperatures [10, 22].

Since minimum temperatures vary greatly across seasons, especially at higher latitudes, critical thermal minimum temperatures (CT_min_) are likely to acclimate substantially. It is known that CT_min_ acclimate [13, 24], that they do so to a greater extent than critical maximum temperatures [12] and that they change more slowly than upper thermal limits [25, 26]. However, the shape and exact duration of this and other acclimation responses is not well studied. Probably the only physiological traits that have been examined with respect to detailed acclimation responses are critical thermal maxima, which acclimate rapidly (within 1–4 days) in some ectotherms [26, 27], and metabolic rates, which acclimate within approximately 14 days in some snakes [28]. Because temperature increases beyond thermal optima rapidly approach lethal limits [7, 24], upper thermal limits may need to acclimate rapidly in many circumstances to allow for immediate survival of sudden short term rises in temperature. Lower temperatures may restrict activity times in colder seasons [15] but are less detrimental in the short term, and critical thermal limits lie far below thermal optima around which individuals thermoregulate [8, 24]. Consequently acclimation of lower thermal limits may be much slower, to match gradual seasonal changes rather than short-term diurnal fluctuations [29]. This may be added to by reduced reaction rates at low temperatures, which likely make slow cold acclimation not only favourable ecologically but also restrict organism’s potential to evolve fast cold acclimation responses [30]. Similarly it is not well known whether tropical organisms use acclimatisation of critical thermal limits to accommodate the comparatively small, yet nevertheless notable, seasonal variations in minimum temperatures. These gaps in our knowledge of acclimation potential and acclimation processes are potentially of great importance, because researchers routinely use critical thermal limits measured under laboratory conditions, and thermal tolerances based on these, as indicators of species’ vulnerability to climate change [7, 31], and as a factor potentially limiting species’ range extents [22, 31] and for other inferences about their physiology [27].

If acclimation of some physiological traits provides a mechanism to adjust gradually to long-term seasonal changes, they are probably completed only after extensive exposure to a specific temperature regime. Experimental acclimation of animals in the laboratory after capture is often not performed at all, or only applied for a widely varying number of weeks (usually anything up to 7 weeks) [6, 12, 24, 32–38]. It has, however, been shown that some ectotherms from climatically different habitats display identical thermal tolerances when acclimated for substantial time periods [39] and that any differences among wild populations would, therefore, result from their extensive acclimation potential rather than from adaptive differences. Many of our estimates of species tolerances may, consequently, be underestimates, or at least highly inconsistent across studies. Because previously published values of thermal tolerances are used extensively in meta-studies [9, 12, 38], understanding the limitations imposed by partial and inconsistent acclimation is crucial. Establishing estimates of the length of time required for different physiological traits to acclimate completely is essential to indicate adequate methodologies, to standardise within and among studies and to assess comparability of values from different studies.

In this study we aimed to describe the time-course and extent of acclimation to cold temperatures in a restricted-range tropical lizard (*Carlia longipes*) and to compare these processes with estimates of average acclimation potential and time required to complete acclimation across reptiles based on published data. We expected that: (i) acclimation responses to cold temperatures can be lengthy and substantial, and are completed in a similar amount of time that gradual temperature trends across seasons would take to manifest; (ii) differences in cold tolerances among reptiles–and potentially in other taxa—observed in the literature are more representative of differences in acclimation time than of true interspecific differences; and (iii) acclimation only occurs if animals are forced into prolonged periods of low temperatures, because acclimation to temperatures that are not representative of true seasonal trends would be disadvantageous. These expectations were confirmed, indicating that lengthy and substantial acclimation may confound results of current meta-studies on thermal limits and vulnerability to climate change.

## Results

**Fig 1:**
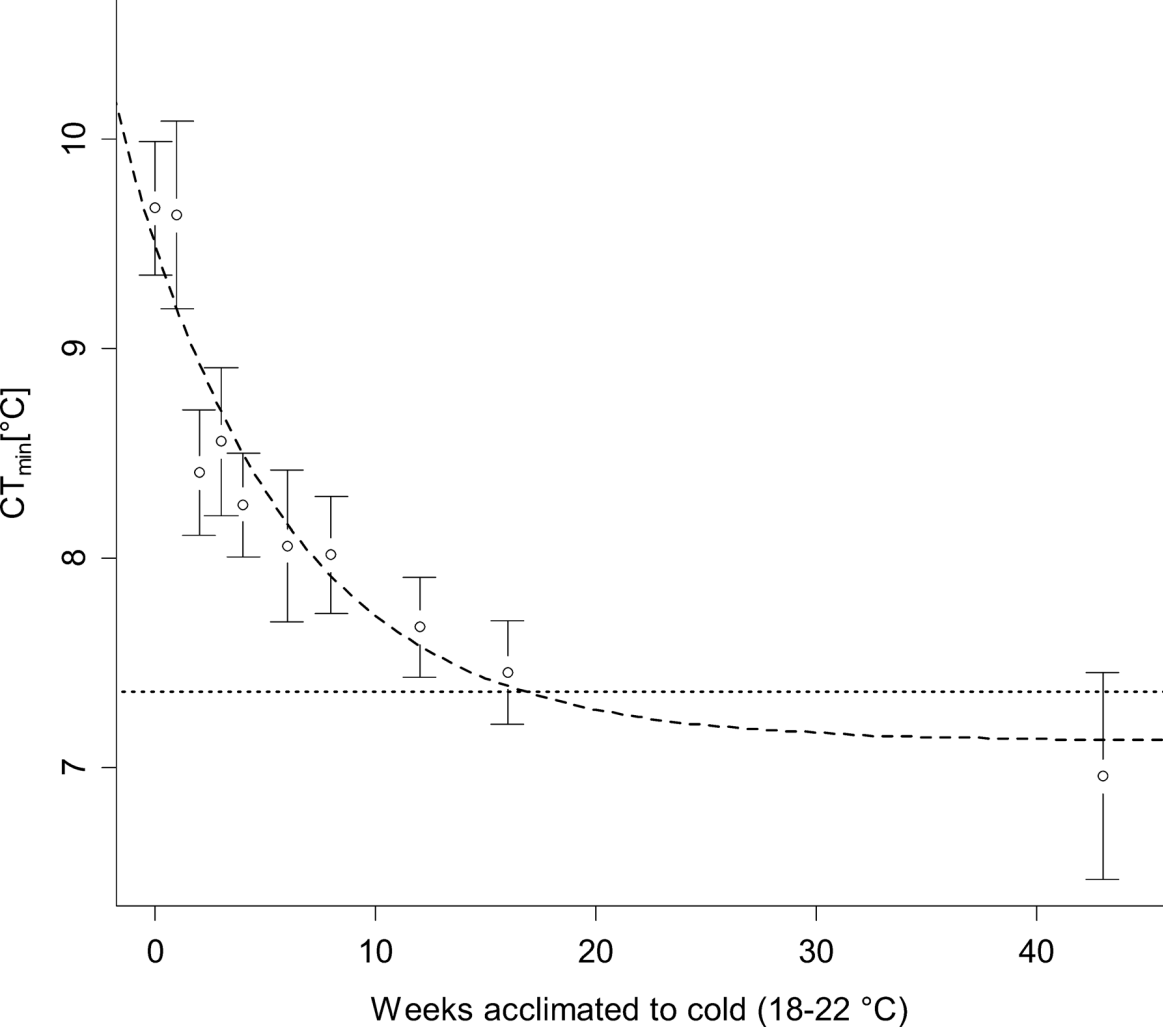
Acclimation of CT_min_ in captive *Carlia longipes*. Critical thermal minimum temperatures [°C± 2SE] of captive individuals of *Carlia longipes* acclimated to a temperature regime of 18–22°C for different time periods measured in weeks. Every week, 3 to 4 previously untested individuals were added to the experimental group and other individuals retested. Past 8 weeks acclimation, only previously tested individuals were used. Predictor line for an average sized animal (4.88g) is shown (dashed). Values approach an asymptote of 7.13°C for an average sized animal, with 95% (7.36°C) of this acclimation completed after 17 weeks (dotted line).

When acclimated to a constant low temperature regime of 18–22°C, CT_min_ in *C*. *longipes* rapidly decreased over the first few weeks and subsequently approached a predicted asymptote of 6.8–7.6°C (depending on body mass), representative of a drop of 2.4°C compared to that of animals collected from the wild (9.67 ± 0.89°C; mean ± SE). The final model describing the acclimation process of CT_min_ in *Carlia longipes* included body mass (ΔAIC = -3.71; χ^2^ = 5.71; p < 0.02; Fig 1) in addition to acclimation time. The CT_min_ of larger animals was, on average, higher than the CT_min_ of smaller animals. The number of times the experiment had been performed on an individual did not significantly affect CT_min_ (ΔAIC = 1.23; χ^2^ = 0.77; p = 0.38).

The acclimation process was best described by the exponential equation

$${CT}_{min}\;\left[{}^{\circ}C\right]=(6.8+0.068*m_{b}\;[g])+2.37\;*\;e^{\left(-0.14\;*\;t_{a}\;[weeks]\right)}$$ (1)

with acclimation time (t_a_) in weeks and body mass (m_b_) in gram as predictor variables, where the expression “6.8 + 0.068 * m_b_” represents the body-mass-specific asymptote. The intra-class correlation coefficient [40, 41], which can lie between values of zero and one and is a measure of how much of the residual variation of the model is accounted for by the random effect, was less than 0.01. This indicates that the random effect explained little of the residual variation. Consequently, the fixed effects explained most of the variation in CT_min_.

**Fig 2:**
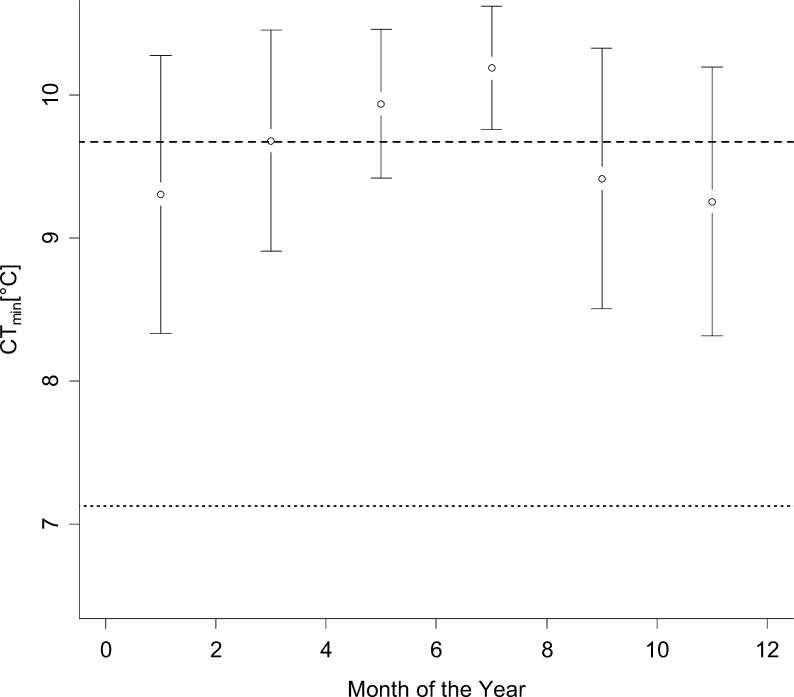
Acclimation of CT_min_ in wild *Carlia longipes*. Critical thermal minimum temperatures [°C ± 2SE] of wild individuals (3 to 8 individuals per group) of *Carlia longipes* collected in different months of a year. No statistically significant differences were observed (ANOVA: F_(5, 25)_ = 1.05, p = 0.4). Lines show mean CT_min_ of wild animals (9.67°C; dashed) and predicted cold acclimated CT_min_ of an average-sized animal of 4.88 g body mass (7.13°C; dotted).

The CT_min_ of individuals of *C*. *longipes* collected in the wild every second month over a one year period did not differ significantly among months (Fig 2; ANOVA: F_(5, 25)_ = 1.05, p = 0.4), although a trend toward a gradual increase in mean CT_min_ was observed from January to July, followed by a similar but opposite trend in CT_min_, with differences of less than 1°C between the highest and lowest mean CT_min_ recorded. Body mass was not significantly correlated with CT_min_ in un-acclimated animals overall (linear regression; F_1,29_ = 0.027, p = 0.87), or if collection month was included as a random effect (F_1,24_ = 0.0021; p = 0.96; linear mixed effects model using R package nlme) [42].

**Fig 3:**
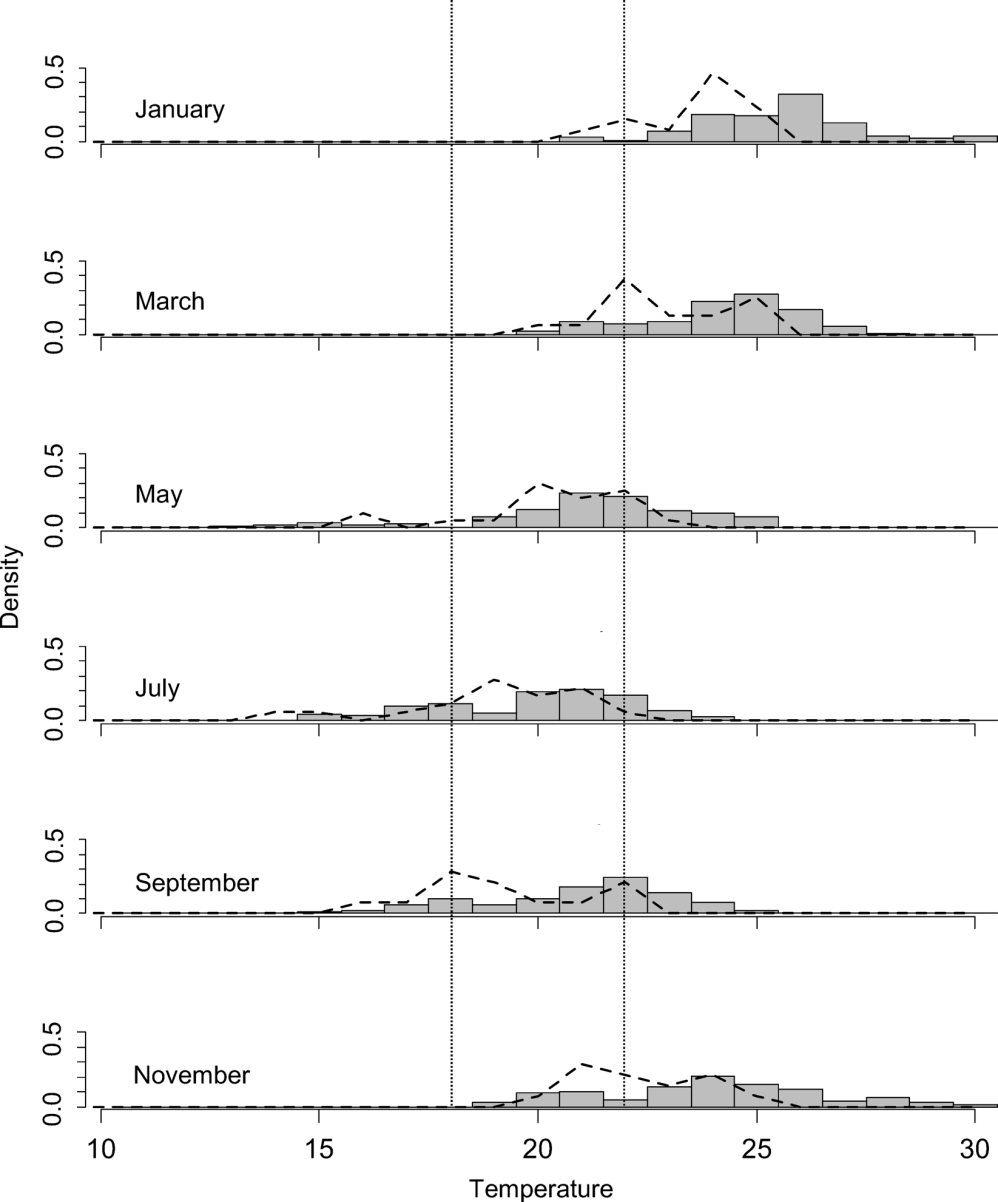
Minimum habitat temperatures of *Carlia longipes*. Density distribution of habitat temperatures (histograms) and minimum temperatures recorded by the Bureau of Meteorology (dashed lines) at different times of year. Habitat temperatures represent records collected at 5 am every day (coldest time of day) over a two-week period in eight different microhabitats representative of the locations that *Carlia longipes* is commonly observed in. Vertical dotted lines show acclimation regime used in this study.

Habitat temperatures available to *C*. *longipes* (Fig 3) at the coldest time of day consistently included higher temperatures than the minimum temperatures recorded by BOM for Cairns over the same time period. In the coldest month recorded for this year, habitat temperatures and minimum temperatures dropped to approximately 14.5°C (14.6 and 14.4°C, respectively) but while BOM minimum temperatures stayed at 22.2°C or lower, experimentally measured habitat temperatures were spread over a broader temperature range and reached 24.4°C (usually under rocks that had been in the sun during the day; pers. obs.). The interquartile range of habitat temperatures in the coldest month at the coldest time of day was 18.2–21.6°C with a median 20.4°C, which is similar to the temperature range of 18–22°C used for experimental acclimation in this study.

**Fig 4:**
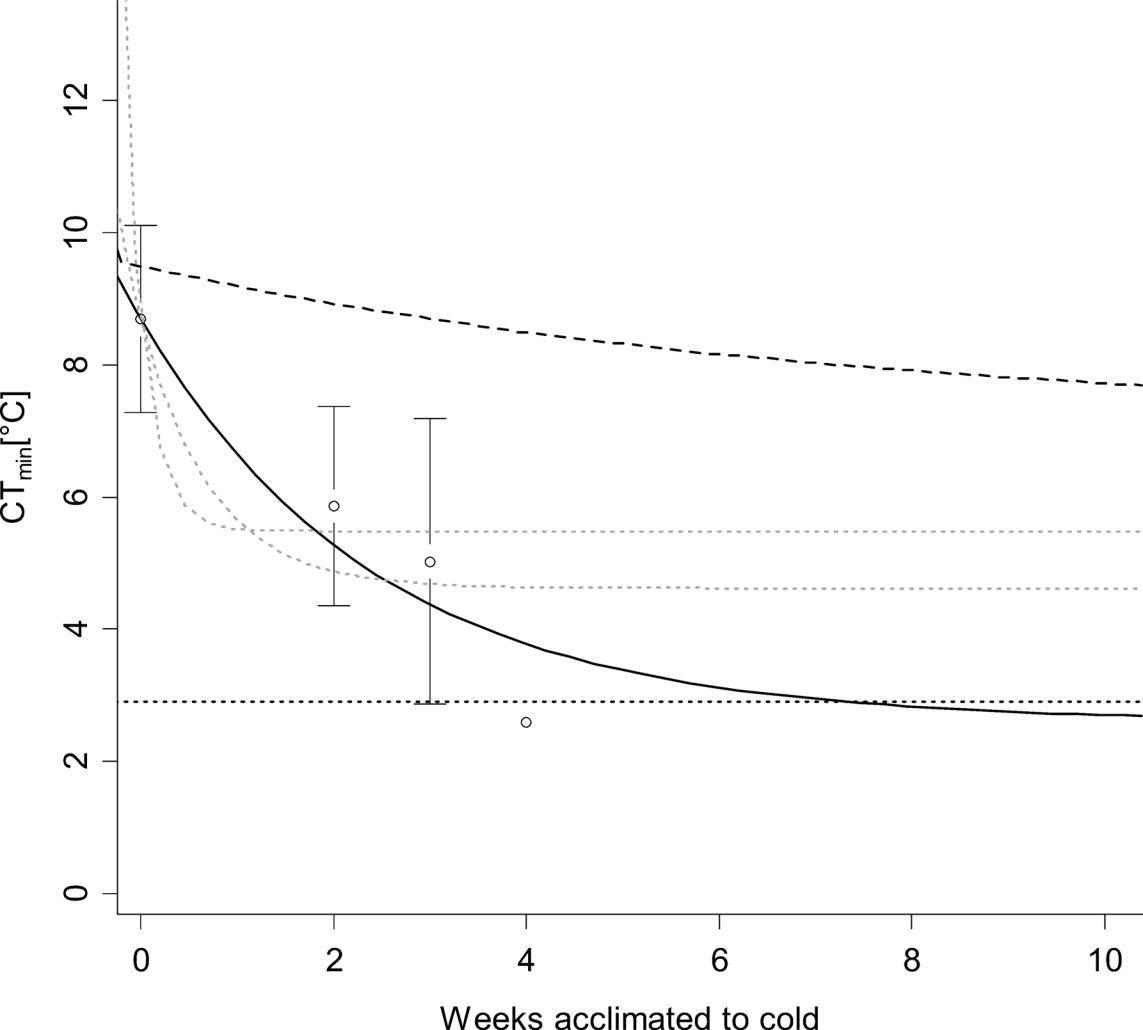
Acclimation of CT_min_ in 22 reptile species. Critical thermal minimum temperatures [°C ± 2SE] of 22 warm acclimated (0 days acclimation) and partially acclimated (2 to 4 weeks acclimation) reptile species. Solid line shows predictions by linear mixed effects model with lowest AIC. Values approach an asymptote of 2.61°C. 95% of acclimation is completed at 7.3 weeks (dotted black reference line). Predictions by other models of inferior fit (dotted black reference line) and acclimation response of *Carlia longipes* (dashed black line) are shown for comparison. The model of best fit was described by the following equation:
$${CT}_{min}\;[{}^{\circ}C]=2.61+6.09*\;e^{\left(-0.41*\;t_{a}\;[weeks]\right)}$$(2)

The average acclimation process of 22 other reptile species, based on the model with the lowest AIC fitted to previously published values of hot acclimated and partially cold acclimated CT_min_ [38] was estimated to be more substantial at 6.1°C but faster, with 95% of the projected acclimation response completed in 7.3 weeks (Fig 4).

It had a significantly lower AIC (304.76) than the other two models based on different asymptote starting values (AIC = 310.65 and 307.60). The final model was highly dependent on starting values for the model asymptote and additional data at longer acclimation times would likely change estimates of final acclimation state. The intra-class correlation was low at 0.02. The model explained 40.2% of the variability of the data, 39.1% of which was explained by acclimation time. The random effect of species ID had a negligible effect on the model fit.

## Discussion

The acclimation response of *Carlia longipes* (Fig 1), as well as that of other reptiles (Fig 4), was substantial, prolonged and explained the bulk of the interspecific differences observed in the literature. True interspecific differences may, therefore, be concealed if data from the literature is used without accounting for differences in acclimation time, because the amount of variation in CT_min_ across acclimation states is substantial compared to the amount of final interspecific differences. This is particularly a problem in meta-analysis because different species are likely to acclimate in different time periods and to different extents, as is apparent from the comparison of the response of *C*. *longipes* to the mean response of other reptiles. Ideal acclimation extent and time for CT_min_ is likely driven by how quickly seasons change in a species’ habitat and by the maximum temperature difference between seasons. Even inclusion of acclimation time as a covariate in meta-analyses [38] may, therefore, be insufficient to account for differences in acclimation time because some species may be fully acclimated at three weeks, while others (such as *C*. *longipes*) take much longer to complete acclimation, i.e. the response cannot be assumed to be comparable among species and cannot be accounted for without detailed knowledge of interspecific differences in acclimation speed and extent.

The acclimation process observed in this study confirmed our predictions that tropical organisms are capable of accommodating environmental changes through substantial physiological changes, despite the relatively stable nature of their habitat. Additionally, their acclimation response may be lengthier than for other organisms, because seasonal changes are slow and cold acclimation is likely avoided unless they are exposed to prolonged low temperature regimes. This is in stark contrast to the rapid acclimation responses in critical thermal maximum temperatures observed in other ectotherms (e.g., some amphibians acclimate in approximately 48 hours) [26]. However, since even short exposures to high temperatures can be lethal, a more immediate response to hot than to cold conditions is not surprising [29].

Critical thermal minimum temperatures in the geographically restricted tropical ectotherm *C*. *longipes* decreased significantly over an extended time period and approached an asymptote approximately 2.4°C below initial values after 17 weeks of continuous acclimation to cold temperatures (Fig 1), with 95% of acclimation completed at 17 weeks after initiation of the acclimation response. Gradual, residual acclimation continued for much longer. This supports our hypotheses that physiological plasticity in at least this tropical organism is stronger than typically appreciated and that acclimatisation as a mechanism should track long-term seasonal changes, rather than short-term temperature fluctuations [29], especially in a habitat where potential for cold acclimatisation may only be advantageous occasionally, in particularly cold years. Records show that minimum annual temperatures in Cairns have occasionally dropped to 6.2°C (coldest record since 1942; CAIRNS AERO at 16.87°S and 145.75°E; http://www.bom.gov.au/climate/ data/ accessed 10.6.2014), which is close to the asymptote of 6.8°C approached by very small individuals of *C*. *longipes* in this study. It is, therefore, likely that the acclimation potential observed here directly reflects selection pressures on populations of *C*. *longipes* over longer periods, especially because tolerance of winter temperatures may be highly relevant in a tropical organism that, as opposed to many temperate organisms, doesn’t avoid such temperatures through complex brumation behaviours [18].

Smaller individuals exhibited lower CT_min_. It has previously been shown that smaller reptiles in some species acclimate more rapidly and to a greater extent [28]. However, in other reptiles smaller individuals have been found to be less cold resistant if not acclimated [43]. Perhaps smaller reptiles require greater tolerance to temperature extremes because they are thermally less buffered, but inconsistent results in the scientific literature warrant further exploration of this possibility, especially since acclimation times applied in that body of literature are inconsistent and short. Naivety of young individuals in selecting thermally optimal microhabitats could also render them more susceptible to environmental temperature fluctuations, making greater absolute tolerance necessary. CT_min_ did not vary with body mass in wild populations, most likely because acclimatisation states in wild animals exposed to different temperatures regimes vary too much to detect statistically significant trends. Further research on the thermal makeup of microhabitats available to or selected by individuals of different sizes within a species may prove valuable to shed light on ontogenetic changes in thermal physiology and thermoregulation.

Regarding thermoregulation, habitat temperatures (Fig 3) show that, in the study year, temperatures only fell to about 14°C in winter, which is a relatively warm winter in Cairns (median minimum annual temperature of 10.2°C, CAIRNS AERO at 16.87°S and 145.75°E; http://www.bom.gov.au/climate/data/ accessed 10.6.2014). Microhabitat temperatures in retreats were centred around a median of 20.4°C with an interquartile range of 18.2–21.6°C, similar to the temperatures used for experimental acclimation in this study. However, these temperatures represent the coldest time of day in the coldest month of the year rather than a continuous exposure. Furthermore, temperatures up to 24.4°C were available to animals in suitable microhabitats, even at this time. Considering that most *Carlia* species thermoregulate behaviourally around preferred body temperatures of 25.5–32.3°C [8], individuals are likely to select higher temperatures throughout the day and may find refuge in the warmest microhabitats available to them at night [44]. Under the conditions observed here, it is therefore highly likely that individuals could, and would, have avoided exposure to low temperatures–and therefore to acclimatisation—whenever possible. This is especially true if this species does not brumate and individuals are attempting to stay active near their optimum temperatures throughout the year. The lack of acclimatisation in wild populations (Fig 2) was very likely a direct result of this, although a slight, non-significant seasonal trend in CT_min_ was observed and larger sample sizes may reveal slight acclimatisation responses in the wild. Stronger decreases in winter temperatures during colder years might nevertheless induce significant acclimatisation responses in wild populations.

Critically, our results suggest that records of temperature tolerance in the literature are most likely underestimates in most cases. Organisms are usually experimentally acclimated for 0–2 weeks and rarely up to 7 weeks [6, 12, 24, 32–38]. If acclimation processes generally follow the time-course we found in *C*. *longipes* (and the best model of acclimation response in reptiles suggests that the period is extended; Fig 4), these acclimation periods are insufficient to capture true tolerances to minimum temperatures. In *C*. *longipes*, 7 weeks would only capture around 62% of the full acclimation potential. Across the broader range of species considered from the literature, at least 7 weeks would be required to complete 95% of acclimation whereas the longest acclimation period of the 22 species for which data was available was 4 weeks. Note also that the form of final models derived was strongly dependent on the starting parameters (see Material and Methods) and we restricted those to similar levels as found in the actual data. More extensive data at longer times would likely extend the period and degree of acclimation represented by the best model here. Generally, measurements of both complete and entirely unacclimated states have relevance if it is acknowledged which of the two is being measured. Unacclimated measures, i.e. CT_min_ of animals immediately after capture will have ecological relevance as measures of susceptibility to extremes of habitat temperatures integrated across their actual (even if not fully defined) recent acclimatisation history, including environmental limits and behavioural responses. Alternatively, lengthy (complete) acclimation in the laboratory provides good estimates of overall physiological tolerances for comparison among species. However, partial acclimation and, especially, comparison of physiological tolerances of species using different degrees of partial acclimation, has little immediate descriptive or predictive relevance, and may disguise patterns of interest. Metastudies using values from studies with greatly varying methodologies regarding acclimation should be aware of this, especially since interspecific variation in CT_min_ has a greater influence on variation in overall thermal tolerance breadth than variation in CT_max_ [11, 12, 22].

If one aims to use CT_min_ values to make inferences on species’ thermal tolerance breadth or potential distribution limits, either prolonged acclimation of study animals or some form of estimate of the expected acclimation response that can be used to predict final acclimation states are necessary for reliable results. Inaccurate inferences, at least in ectotherms, are less likely to be a problem if critical thermal maxima only were used, as the time-course of potential acclimation is rapid [26] and the degree of acclimation is less [13]. However, restricting inferences to critical thermal maxima has limited value, as they vary less geographically [13, 22] and geographic distributions in ectotherms are largely limited by cold tolerances [14–16].

We have shown that acclimation responses can be more substantial and more prolonged than implied by the short and inconsistent time periods used in the literature, while animals in the wild appear to avoid acclimation by appropriate microhabitat selection when possible [45]. In contrast to suggestions in the literature [46], the substantial acclimation potential of *C*. *longipes* suggests that thermoregulation does not preclude their adaptation to low temperatures, despite buffering acclimation responses. Our study is, to our knowledge, one of very few studies describing the shape of the acclimation response for a physiological trait, and the only one examining cold acclimation of lower (rather than upper) thermal limits, over a prolonged time period [26, 47–49]. The extent to which the cold acclimation response depends on acclimation temperatures, diurnal fluctuations in acclimation temperature or diurnal rhythms in photoperiod is not well known [29] and further research in this direction would improve our ability to predict the impacts of long-term changes in environmental conditions. The slow, extensive acclimation abilities observed here need be taken into account when inferences are made based on published values for physiological traits.

## Materials & Methods

### Animal collection and husbandry

*Carlia longipes* is a tropical scincid species restricted to the area between approximately Gordonvale (-17.1°S) and Cooktown (-15.5°S) in Tropical North Queensland, Australia. Overall, 45 individuals were used for the experiments in this study. A first group of individuals of *Carlia longipes* were captured at the end of the warm, wet season (May) 2012, before seasonal decreases in temperature are likely to elicit a cold acclimatisation response. Individuals were housed separately in plastic containers (300 x 200 x 100 mm) with mesh lids on a substrate of commercially available potting soil and leaf litter dried at 60°C. Water was provided ad libitum and food (crickets: *Acheta domestica*) was provided three times per week. Immediately after animal collection, containers were placed in a constant temperature room, with air temperature of 26°C, on aluminium shelving that was cooled at one end by cold water pumped through aluminium tubes. The setup produced a cold acclimation regime with a thermal gradient between the back and the front of each container ranging from 18 to 22°C inside the containers. Body size ranged from 0.68 g to 12.76 g with a mean of 4.88 g.

### Acclimation of critical thermal minimum

At various intervals (0, 1, 2, 3, 4, 6, 8, 12, 16, and 43 weeks) from the start of acclimation, i.e. from the time animals were first subjected to a low temperature regime of 18–20°C, the CT_min_ of a subset of the animals was determined. At intervals between 0 and 8 weeks, CT_min_ was quantified for a previously untested subset of individuals (3 to 4 naïve individuals) as well as for groups that had been tested in any of the previous weeks. This allowed us to assess any potential effects of repeated testing on acclimation. As a result, the number of individuals increased in groups tested at longer acclimation times, with 21 individuals in the final group at 8 weeks acclimation. In week 12 and 16, only these 21 individuals from previously tested groups were tested again, because we originally did not expect acclimation to continue beyond 8 weeks, and therefore did not prepare additional naïve groups for these longer time intervals. A random subset of 12 individuals was re-tested at 43 weeks.

The number of times an individual had been tested did not have a significant effect on the final model (see results) and therefore the lack of naïve groups from the last time intervals (12, 16 and 43 weeks) had no potentially confounding effect on our results. For the “43 week” interval we used a pooled group of animals with acclimation periods beyond 8 months (36 to 49 weeks) with an average acclimation time of 43 weeks. This interval was included to provide an estimate of final CT_min_ (at an interval beyond any seasonal periodicity possibly experienced in the wild).

From May 2012, a new group of 3 to 8 individuals was captured at bimonthly intervals to examine potential acclimatisation of CT_min_ in the wild. Overall, 3 naïve wild individuals were tested in May, 8 in July, 6 in September, 4 in November, 5 in January and 5 in March. Variation in numbers resulted from variation in capture success. CT_min_ of these animals was measured within 24 hours of capture and they were subsequently released. The high abundance of *Carlia longipes* at our capture site at James Cook University, Cairns, made it highly unlikely we would accidentally recapture the same individual in different months.

For CT_min_ experiments, individuals were separately placed in a cylindrical plastic container inside a temperature-controlled cabinet and left for 30 minutes at 18°C, to ensure that all individuals had the same body temperature before the start of the experiments. Following this, the air temperature in the cabinet was cooled, resulting in gradual temperature decrease within the container of approximately 0.2°C per minute, which was continuously recorded with a thermocouple. Animals were flipped on their back every 60 seconds by slowly rolling the container. CT_min_ was defined as the temperature at which loss of righting response occurred, at which point air temperature within the container was recorded. To estimate lizard body temperature from air temperature in the container, a calibration correction was determined by measuring the body temperature of five museum specimens of rainbow skinks of different sizes (range: 0.47 to 10.96g; obtained from a collection at James Cook University) while air temperature in the container was decreased, to establish the relationship between body mass and the time-lag of body temperature behind air temperature at the cooling rate we used. This correction was applied to CT_min_ values of live animals. Body mass in grams was recorded to two decimal places on a laboratory scale. Museum specimens stored in ethanol were rehydrated at 4°C in saline for 3 days before the calibration experiment.

Data on warm acclimated and partially cold acclimated CT_min_ of other reptiles was obtained from a recently published meta-study on acclimation potential of critical thermal limits [38]. We obtained measures of cold acclimated CT_min_, acclimation temperatures used by the cited studies, acclimation time, and acclimation response ratios (ARR) for CT_min_ values. We used the ARR to calculate the warm acclimated CT_min_ of each species (these were not provided in the supplementary material and had to be obtained through calculation). We cross referenced all calculations with the source studies to ensure that values were accurate representations of the original values reported by the cited studies. This gave us two values of CT_min_ for each of 22 reptile species, warm acclimated CT_min_ and CT_min_ at a certain acclimation time.

### Environmental temperatures

Nocturnal (i.e. minimum) microhabitat temperatures for *Carlia longipes* were recorded in each month when naïve wild individuals were collected over a 14 to 20 day period at 5 am (i.e. one measurement per day before sunrise) using Thermochron^®^ iButtons^®^ placed in 8 different locations representative of microhabitats utilised by the species (pers. obs.). These included two locations under leaf litter (one under vegetation and one in the open), two locations 10cm above the leaf litter (one under vegetation and one in the open), two locations in rock crevices (one under a rock that was in the sun during the day and one under a shaded rock), one on top of a rock that was in full sun during the day and one 10cm above the same rock. These locations are representative of microhabitats that individuals are actively foraging in as well as locations for potential refuge with different degrees of temperature buffering compared to ambient air temperatures. Because only nocturnal microhabitat temperatures were being used, there was no need to account for effects of solar radiation on the sensors. The recorded microhabitat temperatures were compared to temperature records from weather stations (CAIRNS AERO at 16.87°S and 145.75°E; http://www.bom.gov.au/climate/data/ accessed 10.6.2014).

### Statistical Analysis

Data was analysed in R 2.13.2 [50]. The acclimation process of *Carlia longipes* was analysed using non-linear mixed effects models (package lme4, function nlmer) [51] to allow for inclusion of animal as a random effect *a priori* to account for the repeated measured design. The model fitted was an exponential decay function of the form

$${CT}_{min}\;[{}^{\circ}C]=A+b\;*\;e^{\left(c*t_{a}\right)}$$ (3)

where A was the asymptote approached as CT_min_ decreased and t_a_ was acclimation time in weeks. This model enabled us to estimate final CT_min_ as the asymptote approached by the response function. The initial model included acclimation time, body mass and number of tests previously performed on each individual (because we used a repeated measures design) as potential predictor variables. The final model was determined by step-wise backwards selection using AIC. The function “nlmer” obtains parameter estimates for the final model of best fit using restricted maximum likelihood. Because naïve wild individuals did not vary in CTmin across seasons, all naïve wild individuals were pooled in the unacclimated starting group at 0 weeks acclimation.

The same function as used for *Carlia longipes* was fitted to the acclimation response of other reptiles [38], but without body mass or number of previous tests as covariates (because they were not reported in the published data set). Final models were highly dependent on starting values used for the estimation of the asymptote of the model (i.e. the estimated final CT_min_), because data was only available for a limited, initial part of the response (i.e. the first three weeks) and only for two time-points per species (one warm acclimated and one partially acclimated value at 14 to 28 days acclimation time). We therefore fitted multiple models with starting values in 1°C intervals between 7°C (the mean CT_min_ of all data points) and -3°C (the mean estimated CT_min_ of all species if the percentage of acclimation completed during their acclimation time was identical to that completed by *Carlia longipes* in the same time). All models converged to one of three different end models. The end model with the lowest AIC was considered the best model and used to describe the estimated, minimum average acclimation response. Species ID was included *a priori* as a random effect in all models. Partial R^2^ of the fixed effects was compared to the overall R^2^ of the final model to assess the how much of the model fit was explained by acclimation time rather than by the random species effect [52].

### Ethics

This research was conducted under the relevant animal ethics approval from the JCU Animal Ethics Committee (Approval Numbers # A1755). Animal collection was conducted under permits from the Queensland Environmental Protection Agency (#WISP10674112).
